# Treatment Effect of Qingfei Paidu Decoction Combined With Conventional Treatment on COVID-19 Patients and Other Respiratory Diseases: A Multi-Center Retrospective Case Series

**DOI:** 10.3389/fphar.2022.849598

**Published:** 2022-07-13

**Authors:** Xingyu Zong, Ning Liang, Jingya Wang, Huizhen Li, Dingyi Wang, Yaxin Chen, Haili Zhang, Liwen Jiao, An Li, Guihui Wu, Jike Li, Mingxuan Wang, Hongde Liu, Zhang Liu, Shusen Zhao, Jin Huang, Qiuhua Huang, Xiaoyan Wang, Jin Qin, Yan Ma, Yanping Wang, Nannan Shi

**Affiliations:** ^1^ Institute of Basic Research in Clinical Medicine, China Academy of Chinese Medical Sciences, Beijing, China; ^2^ Public Health Clinical Center of Chengdu, Chengdu, China; ^3^ Suining Central Hospital, Suining, China; ^4^ Shijiazhuang Fifth Hosipital, Shijiazhuang, China; ^5^ Suihua City First Hospital, Suihua, China; ^6^ Qiqihar Institute for The Prevention and Treatment of Infectious Diseases, Qiqihar, China; ^7^ People’s Hospital of Guangxi Zhuang Autonomous Region, Nanning, China; ^8^ Jinzhong Infectious Disease Hospital, Jinzhong, China; ^9^ Beijing University of Chinese Medicine, Beijing, China

**Keywords:** COVID-19, respiratory diseases, Qingfei Paidu decoction, TCM, case series

## Abstract

**Background:** Qingfei Paidu decoction (QFPDD) has been widely used in treating coronavirus disease 2019 (COVID-19) in China. However, studies on the treatment effect of COVID-19 patients and other respiratory diseases have not been well demonstrated. Our study aims to determine the treatment effect of QFPDD in combination with conventional treatment on COVID-19 patients and other respiratory diseases.

**Methods:** This retrospective study recruited COVID-19 patients who were treated with QFPDD for at least two courses (6 days) from seven hospitals in five provinces from January 21 to March 18 2020. Demographic, epidemiological, clinical, laboratory, computed tomography characteristics, treatment, and outcome data were collected and analyzed. The improvements in clinical symptoms before and after QFPDD treatment were compared.

**Results:** Eight COVID-19 patients were included in this study. Of them, six were males (75.0%). The median age of the patients was 66 (60–82) years. Four patients were classified as mild and moderate cases (50.0%); there were two severe cases (25.0%) and critical cases (25.0%). The most common symptom was cough (7 [87.5%]), followed by fever (6 [75.0%]), fatigue (4 [50.0%]), asthma (4 [50.0%]), and anorexia (3 [37.5%]). Abnormal findings included decrease in neutrophils (3 [37.5%]), lymphocytes (2 [25.0%]), alkaline phosphatase (3 [37.5%]), lactic dehydrogenase (4 [50.0%]), erythrocyte sedimentation rate (2 [25.0%]), and C-reactive protein (5 [83.3%]) at admission. After one course (3 days) of QFPDD, nasal obstruction and sore throat completely disappeared, and fever (5 [83.3%]), fatigue (2 [50.0%]), and cough (2 [28.6%]) were improved. After two courses (6 days), the fever disappeared completely in all patients, and the other symptoms showed a tendency to improve. In non-severe patients, 87.5% baseline symptoms completely disappeared. In severe patients, 61.1% of the baseline symptoms completely disappeared after patients were administered QFPDD for two courses. Of the abnormal indicators, 55.6% returned to normal levels. The median duration to complete fever recovery was 1.0 day. The median durations of viral shedding and hospitalization were 10.5 and 21.5 days, respectively. None of the patients worsened and died, and no serious adverse events occurred related to QFPDD during hospitalization.

**Conclusion:** QFPDD combined with conventional treatment improved clinical symptoms in COVID-19 patients with other respiratory diseases, and no serious adverse reactions associated with QFPDD were observed. Larger sample studies confirm our findings in the future.

## Introduction

Coronavirus disease 2019 (COVID-19) is an acute respiratory infection caused by the novel coronavirus (severe acute respiratory syndrome coronavirus 2) and has become a global health emergency (Ma et al., 2021; Zhu et al., 2020). As of November 30 2021, approximately 260 million confirmed cases of COVID-19 and approximately 5.2 million cumulative deaths have been reported worldwide (World Health Organization, 2021). Sustainable and effective strategies are urgently required to overcome this pandemic. Presently, the pandemic in China has been effectively controlled using multiple strategies implemented by the Chinese government, including the implementation of traditional Chinese medicine (TCM) in combination with conventional treatment in clinical practice. TCM has shown favorable effects in previous public health emergencies of international concern, such as severe acute respiratory syndrome and Middle East respiratory syndrome (Zhang et al., 2004). In addition, it also had inspiring effects on the treatment of COVID-19, and early treatment with Qingfei Paidu decoction (QFPDD) was associated with favorable outcomes (Shi et al., 2020; Ma et al., 2020). Moreover, two systematic reviews and meta-analyses demonstrated the effectiveness and safety of QFPDD in treating COVID-19 patients (Wang J. et al., 2021; Zhang et al., 2022). Since the sixth version of the national guideline Diagnosis and Treatment Protocol for Novel Coronavirus Pneumonia has been issued, QFPDD has been recommended as a general formula for the treatment of all COVID-19 patients including mild, moderate, severe, and critically ill cases.

A recent study has shown that COVID-19 cases combined with other respiratory diseases account for 2.3% of all cases, and patients with combined diseases are more likely to have a worse prognosis than patients without combined diseases (Ma et al., 2021). Another study also found that patients with COVID-19 and other respiratory diseases, such as chronic obstructive pulmonary disease (COPD), are more susceptible to exacerbation and impaired lung function caused by the virus, leading to severe outcomes and higher mortality rates (Higham et al., 2020; Tadolini et al., 2020). Therefore, effective management of COVID-19 patients with complications of other respiratory diseases is required.

To date, few studies have detailed the clinical characteristics, laboratory and computed tomography funings, and treatment outcomes of COVID-19 patients with other diseases of the respiratory system. To further explore the effectiveness of QFPDD, we aimed to retrospectively analyze the data of patients treated with QFPDD combined with conventional treatment in seven hospitals in five provinces from January 21 to March 18 2020. The findings of this study will provide a reference for integrating TCM into the management of COVID-19.

## Materials and Methods

Eight patients with confirmed COVID-19 and other respiratory diseases who were treated with QFPDD in combination with conventional treatment were enrolled from seven hospitals in five provinces between January 21 and March 18 2020. The inclusion criteria were as follows: 1) laboratory-confirmed COVID-19 cases; 2) comorbid with other respiratory diseases, including but not limited to bronchiectasis, COPD, tuberculosis, lobar pneumonia, chronic bronchitis, and emphysema; and 3) administered with at least two courses (6 days) of QFPDD.

The diagnostic criteria for COVID-19 were based on the *Diagnosis and Treatment Guidelines for 2019 Novel Coronavirus Pneumonia* (referred to as *the Guidelines*) issued by the National Health Commission of the People’s Republic of China (The NHC of the People’s Republic of China, 2020)*.* According to *the Guidelines*, the patients were classified as mild, moderate, severe, and critical cases. Mild cases were defined as those with mild clinical symptoms and no pneumonia was observed on imaging. Moderate cases were defined as those with fever, respiratory symptoms, or imaging manifestations of pneumonia. Severe cases were defined as those meeting any of the following criteria: 1) shortness of breath, respiratory rate ≥ 30 times/min; 2) oxygen saturation ≤93% at rest; and 3) arterial partial pressure of oxygen (PaO_2_)/fraction of inspired oxygen (FiO_2_)≤300 mmHg (1 mmHg = 0.133 kPa). Critical cases were defined as patients with respiratory failure requiring mechanical or noninvasive ventilation, shock, or combined with other organ failure, requiring intensive unit care. We further categorized mild and moderate patients as non-severe cases and severe and critical patients as severe cases. We defined the time to clinical symptom resolution as the time from using QFPDD to the first clinical symptom resolution, lasting at least 24 h. Absorption of lung lesions refers to the absorption of lung lesions shown by lung imaging at discharge.

In the present study, all patients were treated with QFPDD combined with conventional treatment. QFPDD comprises 21 herbs. Chinese, English, and Latin names and the dose of these herbs are shown in Table 1. Conventional treatment includes antiviral drugs (alpha interferon, ribavirin, arbidol, oseltamivir, lopinavir–ritonavir, and Coolidge), antibiotics (cefuroxime, cefoperazone, ceftriaxone sodium, piperacillin sodium, tazobactam sodium, voriconazole, and moxifloxacin), and symptomatic treatments. The decoction was prepared according to the formula and the quality standards of the 2015 Chinese Pharmacopoeia.

**TABLE 1:** Composition of QFPDD.

Chinese name	Pinyin Name	TCM name	English name	Latin name	Dose (g)	Tissue used
麻黄	Ma Huang	*Ephedrae Herba*	Chinese ephedra herb	*Ephedra sinica Stapf* (Ephedraceae)	9	Herbaceous stem
细辛	Xi Xin	*Asari Radix et Rhizoma*	Manchurian wild ginger herb	*Asarum heterotropoides F.Schmidt* (Aristolochiaceae)	6	Roots and rhizome
桂枝	Gui Zhi	*Cinnamomi ramulus*	Cassia bark tree twig	*Cinnamomum cassia (L.) J.Presl* (Lauraceae)	9	Young shoot
广藿香	Guang Huo Xiang	*Pogostemonis Herba*	Cablin patchouli herb	*Agastache rugosa* (*Fisch. and C.A.Mey.*) *Kuntze* (Lamiaceae)	9	Aerial part
白术	Bai Zhu	*Atractylodis Macrocephalae Rhizoma*	Largehead atractylodes rhizome	*Atractylodes macrocephala Koidz.* (Asteraceae)	9	Rhizome
山药	Shan Yao	*Dioscoreae Rhizoma*	Common yam rhizome	*Dioscorea oppositifolia L.* (Dioscoreaceae)	12	Rhizome
石膏	Shi Gao	*Gypsum Fibrosum*	Plaster Stone	*Gypsum Fibrosum*	15	-
柴胡	Chai Hu	*Bupleuri Radix*	Chinese thorowax root	*Bupleurum chinense DC. (*Apiaceae*)*	16	Roots
苦杏仁	Ku Xing Ren	*Armeniacae Semen Amarum*	Ansu apricot seed	*Prunus amygdalus Batsch* (Rosaceae)	9	Seed
款冬花	Kuan Dong Hua	*Farfarae Flos*	Common coltsfoot flower	*Tussilago farfara L.* (Asteraceae)	9	Flower
紫菀	Zi Wan	*Asteris Radix et Rhizoma*	Tatarian aster root and rhizome	*Aster tataricus L.f.* (Asteraceae)	9	Roots
射干	She Gan	*Belamcandae Rhizoma*	Blackberry rhizome	*Iris domestica (L.) Goldblatt and Mabb.* (Iridaceae)	9	Rhizome
半夏	Ban Xia	*Pinelliae Rhizoma Cum Zingibere et Alumine*	Ternate pinellia	*Pinellia ternata (Thunb.) Makino* (Araceae)	9	Tuber
生姜	Sheng Jiang	*Zingiberis Rhizoma Recens*	Fresh ginger	*Zingiber officinale Roscoe* (Zingiberaceae)	9	Rhizome
枳实	Zhi Shi	*Aurantii Fructus Immaturus*	Immature bitter orange	*Citrus × aurantium L.* (Rutaceae)	6	Immature fruit
陈皮	Chen Pi	*Citri Reticulatae Pericarpium*	Tangerine peel	*Citrus tangerina Yu.Tanaka* (Rutaceae)	6	Fruit peel
猪苓	Zhu Ling	*Polyporus*	Agaric	*Polyporus*	9	Sclerotium
茯苓	Fu Ling	*Poria*	Indian buead tuckahoe	*Poria*	15	Sclerotium
泽泻	Ze Xie	*Alismatis Rhizoma*	Oriental water plantain tuber	*Alisma plantago-aquatica subsp. orientale* (*Sam.*) *Sam.* (Alismataceae)	9	Tuber
黄芩	Huang Qin	*Scutellariae Radix*	Baikal skullcap root	*Scutellaria baicalensis Georgi* (Lamiaceae)	6	Roots
炙甘草	Zhi Gan Cao	*Glycyrrhizae Radix et Rhizoma Praeparata Cum Melle*	Liquorice root	*Glycyrrhiza glabra L.* (Fabaceae)	6	Roots and rhizome

We retrospectively collected demographic, epidemiological, clinical, laboratory, computed tomography characteristics, treatment, and outcome data from medical records. The eligibility of the patients for this study and their data were independently checked and extracted by two researchers. If the core data, such as clinical, laboratory and CT findings, and treatment outcomes were unclear or missing, we would contact doctors in charge of the patients to supplement the data as soon as possible.

Adverse events included nausea, vomiting, and abdominal pain. If symptoms are relieved after stopping QFPDD treatment, this adverse event may be related to QFPDD. If an adverse reaction persists after stopping QFPDD treatment, then it is less likely to be associated with QFPDD.

Continuous measurements as median (IQR) or mean (SD) is taken accordingly to know whether they are normally distributed or not. Categorical variables were described as frequency and percentages. All statistical analyses were performed with SPSS software, version 13.0 (SPSS Inc.).

## Results

### Case Series

Case 1 involved a 62-year-old man with mild COVID-19 combined with hypertension, diabetes, and bronchiectasis. He had symptoms of a sore throat. After admission to the hospital on February 7 2020, he was administered with interferon alpha, arbidol, lopinavir, and other antivirals, and cefuroxime, cefoperazone, two antibiotics, and glucocorticoids. But the symptoms of sore throat were not relieved significantly. Three days after admission, several TCM doctors were invited to participate in the consultation of this patient, and then he was treated with one dose of QFPDD three times a day. Five days after admission, his sore throat improved. The nucleic acid test result turned negative. Computed tomography (CT) results of the lungs showed no exudation or signs of pneumonia, and no serious adverse events were observed. Nine days after administration, his condition further improved, and he was discharged from the hospital on February 16 2020.

Case 2 was a 66-year-old woman with moderate COVID-19, hypertension, coronary heart disease, and diabetes. She visited the hospital because of cough, sputum, blood in the sputum, and fever. After admission on February 5 2020, as the patient’s symptoms of cough did not improve, it was thought suitable to administer QFPDD through a joint consultation including TCM doctors. Therefore, in addition to taking lopinavir–ritonavir and arbidol, she was administered one dose of QFPDD three times a day. After one course of the treatment (3 days), symptoms of fever, anorexia, and nasal obstruction improved. After 7 days of QFPDD treatment, her cough was completely cured. After administering QFPDD for 23 days, the nucleic acid test result became negative. No serious adverse events occurred during the treatment, and the patient was discharged on March 10 2020.

Case 3 was a 67-year-old woman with moderate COVID-19 and tuberculosis. She was admitted to the hospital on February 2 2020 with symptoms of cough. After group consultations with doctors practicing Chinese and Western medicine, she was treated with one dose of QFPDD three times a day. In addition, we administered interferon alpha and arbidol. After taking QFPDD for 2 days, her symptoms of cough significantly improved, and the nucleic acid test result turned negative after admission. Furthermore, serious adverse events were not observed. She recovered and was discharged on February 19 2020.

Case 4 involved a 72-year-old man with moderate COVID-19 and tuberculosis. He was admitted to the hospital on January 22 2020, and presented with fever and cough. The symptoms were recurrent after conventional treatment and there was no significant improvement. After inviting doctors of TCM to participate in the consultation, he was advised to be treated with one dose of QFPDD three times a day. After taking QFPDD, the fever was quickly relieved. The cough was completely cured after 4 days of TCM treatment. Sixteen days after admission, the symptoms significantly improved and the nucleic acid test result was negative. Serious adverse events were not observed. The patient recovered and was discharged on February 14 2020.

Case 5 involved a 60-year-old man with severe COVID-19 who also had type I respiratory failure and community-acquired pneumonia. He was admitted on February 15 2020 and presented with fever, fatigue, cough, and asthma. Once he was admitted, doctors held a group consultation with doctors practicing Chinese and Western medicine to decide whether to use QFPDD. After the evaluation by TCM doctors, he was treated with one dose of QFPDD three times a day. Seven days after admission, the symptoms significantly improved, and the nucleic acid test result was negative. On February 24, chest CT showed improvement in the lung lesions. No serious adverse events occurred during the treatment. The patient was cured and discharged on February 26 2020.

**FIGURE 1:**
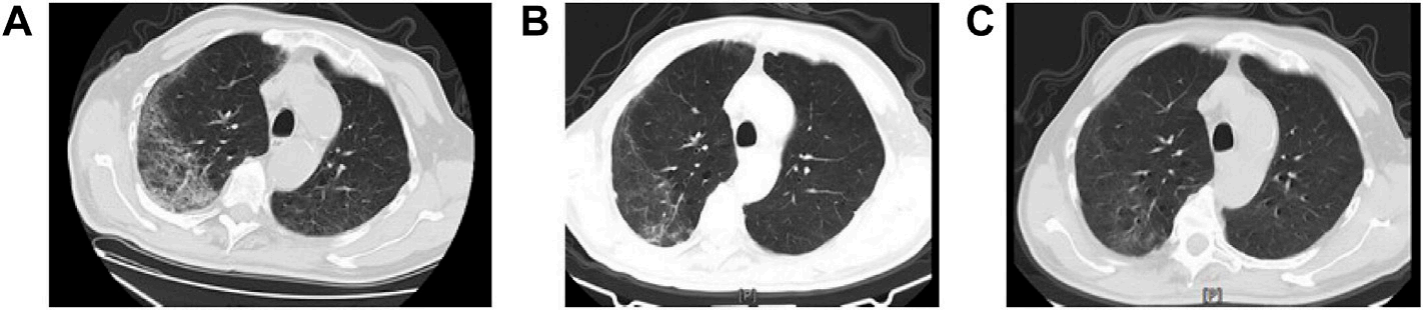
Chest computed tomographic (CT) images of a 66-year-old COVID-19 patient before and after QFPDD. Case 6: A 66-year-old male with severe COVID-19. Panel 2-**(A)**: In both lungs, transmissibility was reduced, and ground-glass opacity and mesh shadows were observed mostly under the pleura and with interstitial involvement. Scattered patchy opacities were observed mainly in the lower lobe of both lungs, and scattered small nodules were present in both lungs. Panel 2-**(B)**: After two courses of QFPDD in combination with conventional treatment, the scattered ground-glass, strip-like, and patchy opacities in both lungs were absorbed and reduced compared to that measured at admission. Panel 2-**(C)**: Ground-glass opacity was further reduced in both lungs before discharge.

Case 6 involved a 66-year-old man with severe COVID-19 and lobar pneumonia. He was admitted to the hospital on February 16 2020 and presented with fatigue, anorexia, and asthma. The doctors held a group consultation with doctors practicing Chinese and Western medicine to decide whether to use QFPDD. This patient was treated with one dose of QFPDD three times a day after admission. After taking two courses of QFPDD, the symptoms were quickly relieved; scattered ground glass, strip, and dot film shadows appeared on both sides of the lung, and the absorption of lung lesions was reduced compared with that measured on the day of admission. Serious adverse events were not observed. We compared chest CT images before and after taking QFPDD (Figure 1). After 29 days of treatment, the symptoms significantly improved and the nucleic acid test result became negative. The patient was discharged on March 18.

Case 7 involved a 65-year-old man in critically ill condition. The patient exhibited a fever of unknown origin, accompanied by cough, paroxysmal dry cough, fatigue, and asthma. On February 16 2020, he was transferred to the hospital because of fever and cough for more than 20 days. No significant improvement was observed after conventional treatment. On February 21, relevant experts and TCM doctors were organized for joint consultation and decided to administer QFPDD. After QFPDD administration, the symptoms rapidly improved, and chest CT showed partial absorption on February 25. Serious adverse events were not observed. The patient was cured and discharged on March 8 2020.

Case 8 was an 82-year-old man who was critically ill. On February 8 2020, he was admitted to the hospital because of a cough for 8 days and fever for 7 days, accompanied by chills, sore throat, muscle soreness, fatigue, and other discomforts. Respiratory diseases, including respiratory failure, chronic bronchitis with emphysema, and electrolyte disorders, were observed. Chest CT showed scattered ground glass and mesh shadows in lungs, subpleural distribution, interstitial involvement, and scattered strip shadows in both lungs, mainly in the lower lobe. He was considered critically ill on February 11. After treatment, his airtightness was relieved and his blood oxygen saturation was approximately 97%. Interferon and acetylcysteine were administered, and the 13-day treatment had no significant effect on chest CT. On February 21, relevant experts and TCM doctors were organized for a consultation and decided to administer QFPDD after discussion. After two courses (February 27, 19 days after admission), symptoms such as cough and asthma were significantly reduced, and the nucleic acid test result turned negative. Before being discharged, chest CT showed scattered spots, plaques, ground glass, and cable shadows in the multi-lobar segments of both lungs, which were much better than those measured before admission. No serious adverse events occurred during QFPDD administration, and the patient was discharged on March 10 2020.

**FIGURE 2:**
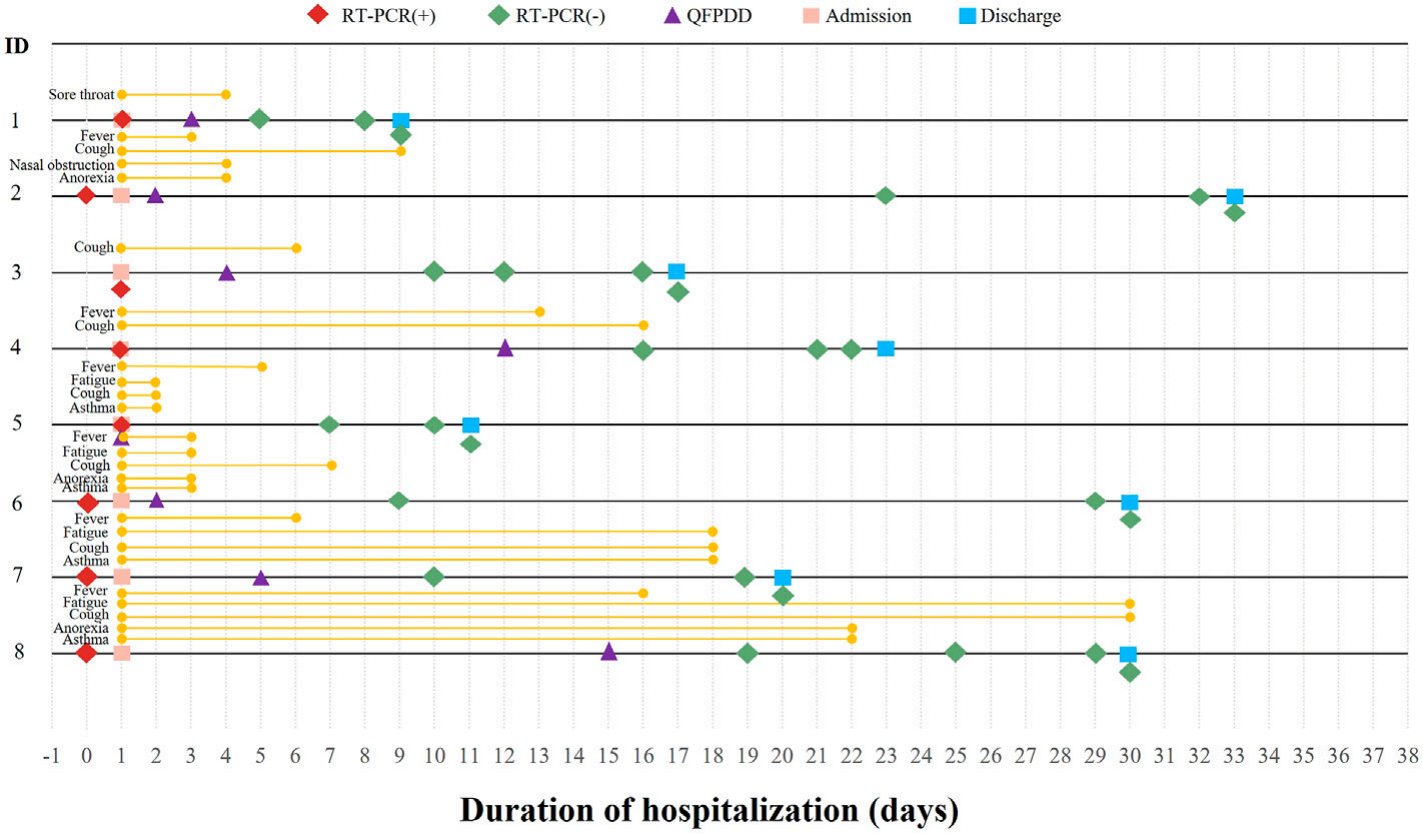
Detailed course of eight COVID-19 cases during hospitalization.

**FIGURE 3:**
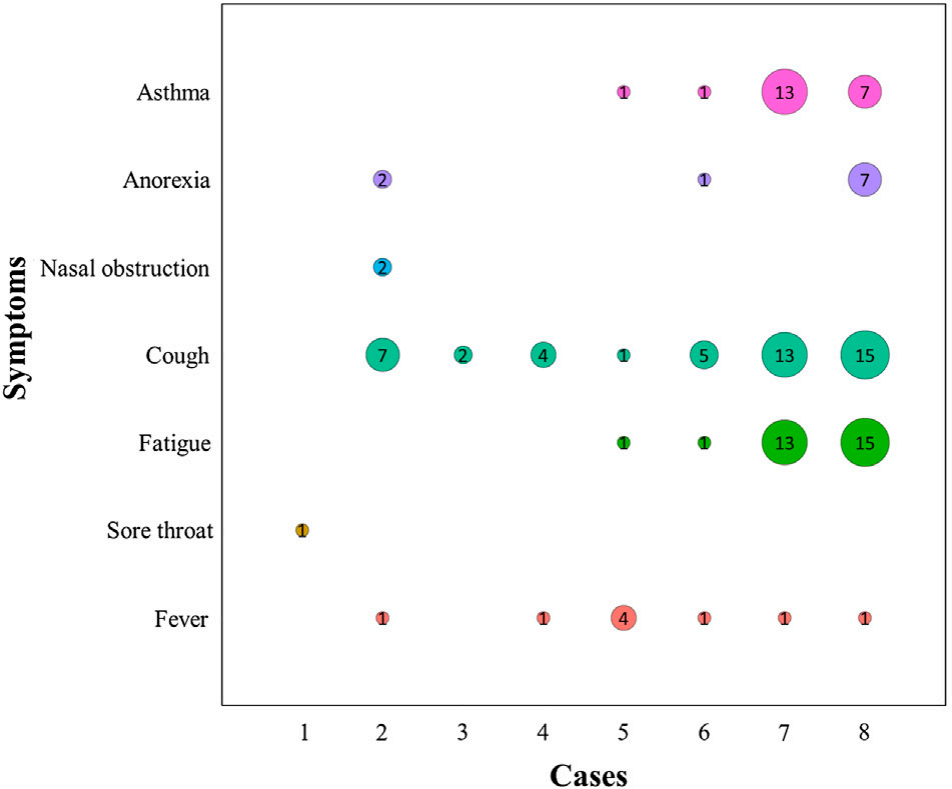
Duration of the major clinical symptom recovery in eight COVID-19 cases. Numbers indicate the days when the major clinical symptoms (asthma, anorexia, nasal obstruction, cough, fatigue, sore throat, and fever) were completely cured. The size of the circle is positively correlated with the number of days.

Detailed information of the aforementioned eight cases is shown in Figure 2, Figure 3, Table 2, and Table 3.

**TABLE 2:** Demographics and clinical characteristics of the eight COVID-19 cases with other respiratory diseases.

Variables	Case 1	Case 2	Case 3	Case 4	Case 5	Case 6	Case 7	Case 8
Age	62	66	67	72	60	66	65	82
Sex	Male	Female	Female	Male	Male	Male	Male	Male
Recently visited Wuhan, China	No	Yes	No	Yes	No	No	No	No
Exposure to source of transmission within the past 14 days	Yes	Yes	No	No	Yes	No	No	No
Date of admission	2020.02.07	2020.02.05	2020.01.31	2020.01.21	2020.02.15	2020.02.16	2020.02.16	2020.02.08
Date of positive nucleic acid test result	2020.02.05	2020.02.03	2020.02.01	2020.01.22	2020.02.14	2020.02.15	2020.01.29	2020.02.07
Clinical classification	Mild	Moderate	Moderate	Moderate	Severe	Severe	Critical	Critical
Other respiratory diseases	1. COPD	1. COPD	1. Tuberculosis	Tuberculosis	1. Community acquired pneumonia*	Lobar pneumonia	1. Bacterial pneumonia	1. Bronchitis
	2. Bronchiectasis	2. Respiratory failure	2. Lobar pneumonia		2. Respiratory failure		2. Fungal pneumonia	2. Emphysema
	—	3. Emphysema	—		—		3. Respiratory failure	3. Respiratory failure
	—	4. Pulmonary cyst	—		—		—	—
Symptoms before QFPDD								
Fever	No	Yes	No	Yes	Yes	Yes	Yes	Yes
Cough	No	Yes	Yes	Yes	Yes	Yes	Yes	Yes
Sore throat	Yes	No	No	No	No	No	No	No
Fatigue	No	No	No	No	Yes	Yes	Yes	Yes
Asthma	No	No	No	No	Yes	Yes	Yes	Yes
Anorexia	No	Yes	No	No	No	Yes	No	Yes
Nasal obstruction	No	Yes	No	No	No	No	No	No
Diarrhea	No	No	No	No	No	No	No	No
Rhinorrhea	No	No	No	No	No	No	No	No
Pharyngula	No	No	No	No	No	No	No	No

Note: *: Pathogens were not clear. COPD: chronic obstructive pulmonary disease.

**TABLE 3:** Treatment medicines, clinical symptoms after QFPDD, and treatment outcomes of eight COVID-19 cases.

Variables	Case 1	Case 2	Case 3	Case 4	Case 5	Case 6	Case 7	Case 8
Treatment medicines								
QFPDD	Yes	Yes	Yes	Yes	Yes	Yes	Yes	Yes
Antiviral treatment^#^	Yes	Yes	Yes	Yes	Yes	Yes	Yes	Yes
Antibiotics^#^	No	No	No	Yes	No	Yes	Yes	No
Corticosteroids	No	No	No	Yes	No	No	Yes	No
Symptoms after one course (3 days) of QFPDD								
Body temperature, °C	36.5	36.3	37.0	36.8	38.3	36.5	36.8	36.0
Cough	No	Yes	Yes	Yes	No	No	No	Yes
Sore throat	No	No	No	No	No	No	No	No
Fatigue	No	No	No	No	No	No	No	Yes
Asthma	No	No	No	No	No	No	No	Yes
Anorexia	No	No	No	No	No	No	No	Yes
Diarrhea	Yes	No	No	No	No	No	No	No
Nasal obstruction	No	No	No	No	No	No	No	No
Rhinorrhea	No	No	No	No	No	No	No	No
Pharyngula	No	No	No	No	No	No	No	No
Symptoms after two courses (6 days) of QFPDD								
Body temperature, °C	No	36.3	37.0	No	37.0	36.3	36.9	36.5
Cough	No	Yes	No	No	No	No	Yes	Yes
Sore throat	No	No	No	No	No	No	No	No
Fatigue	No	No	No	No	No	No	Yes	Yes
Asthma	No	No	No	No	No	No	Yes	Yes
Anorexia	No	No	No	No	No	No	No	Yes
Diarrhea	No	No	No	No	No	No	No	No
Nasal obstruction	No	No	No	No	No	No	No	No
Rhinorrhea	No	No	No	No	No	No	No	No
Pharyngula	No	No	No	No	No	No	No	No
Treatment outcomes								
Date of the first negative RT-PCR tested	2020.02.12	2020.02.28	2020.02.12	2020.02.07	2020.02.20	2020.03.17	2020.02.17	2020.02.27
Duration of viral shedding (days)	5	24	10	16	5	10	11	20
Duration of fever recovery (days)	—	1	—	1	4	1	1	1
Date of discharge	2020.02.16	2020.03.10	2020.02.19	2020.02.14	2020.02.26	2020.03.18	2020.03.08	2020.03.10
Duration of hospitalization (days)	9	33	17	23	11	30	20	30
Worsened	No	No	No	No	No	No	No	No

#Antiviral agents include alpha interferon, ribavirin, arbidol, oseltamivir, lopinavi–ritonavir, and Coolidge.

# Antibiotics include cefuroxime, cefoperazone, ceftriaxone sodium, piperacillin sodium, tazobactam sodium, voriconazole, and moxifloxacin.

### Demographic and Baseline Characteristics

A total of eight patients (six men and two women) were included in this study, and their demographic and baseline characteristics are given in Table 2. The median age of the patients was 66 (range 60–82). Four patients were classified as mild and moderate cases (50.0%); two patients, severe cases (25.0%); and two patients, critical cases (25.0%). Other accompanying respiratory diseases included respiratory failure and acute respiratory failure (acute respiratory distress syndrome) (3 [37.5%]), COPD (2 [25.0%]), tuberculosis (2 [25.0%]), lobar pneumonia (2 [25.0%]), bronchiectasis (1 [12.5%]), bronchitis (1 [12.5%]), emphysema (1 [12.5%]), bacterial pneumonia (1 [12.5%]), and fungal pneumonia (1 [12.5%]). The symptoms with the highest frequency before QFPDD administration were cough (7 [87.5%]), fever (6 [75.0%]), fatigue (4 [50.0%]), asthma (4 [50.0%]), and anorexia (3 [37.5%]).

### Clinical Symptom Improvements and Treatment Outcomes

**FIGURE 4:**
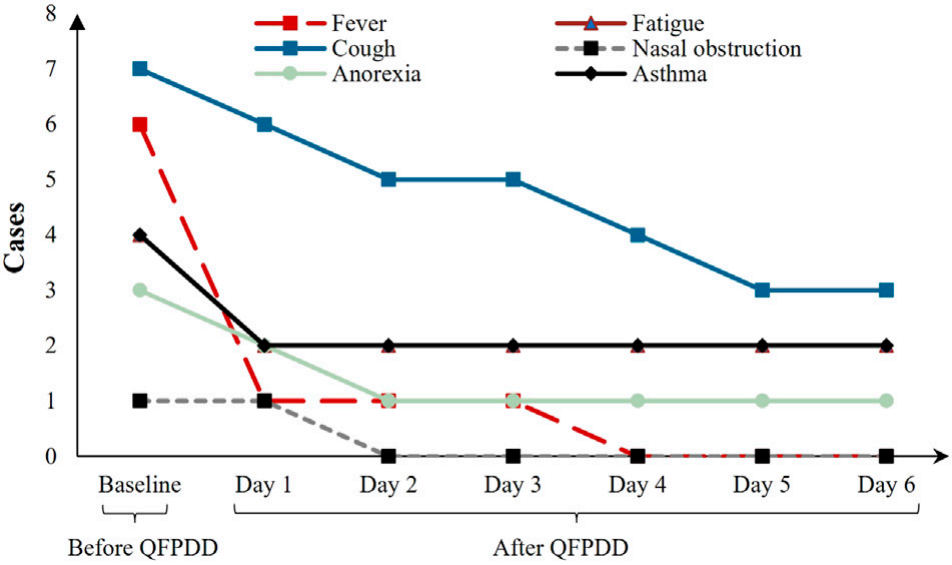
Longitudinal course of clinical symptoms in eight COVID-19 cases.

The trend of clinical symptom improvements in the eight patients during the two courses of QFPDD treatment is shown in Figure 4. Throughout the treatment process, the symptoms of fever, fatigue, cough, nasal obstruction, anorexia, and asthma improved to varying degrees after QFPDD administration (Figure 3).

After one course of QFPDD (3 days), nasal obstruction and sore throat were completely cured, and clinical symptoms such as fever (5 [83.3%]), fatigue (2 [50.0%]), and cough (2 [28.6%]) improved. After two courses (6 days), the fever disappeared completely in all patients, and the other symptoms showed a tendency to improve. In non-severe patients, 87.5% baseline symptoms completely disappeared. In severe patients, 61.1% of the baseline symptoms completely disappeared after patients were administered QFPDD for two courses. Of the abnormal indicators, 55.6% returned to normal levels. In addition to QFPDD, most patients received antiviral treatment, among which five (62.5%) received antibiotics and two (25.0%) received corticosteroids. The treatment process and related information for the eight patients are presented in Table 3.

**FIGURE 5:**
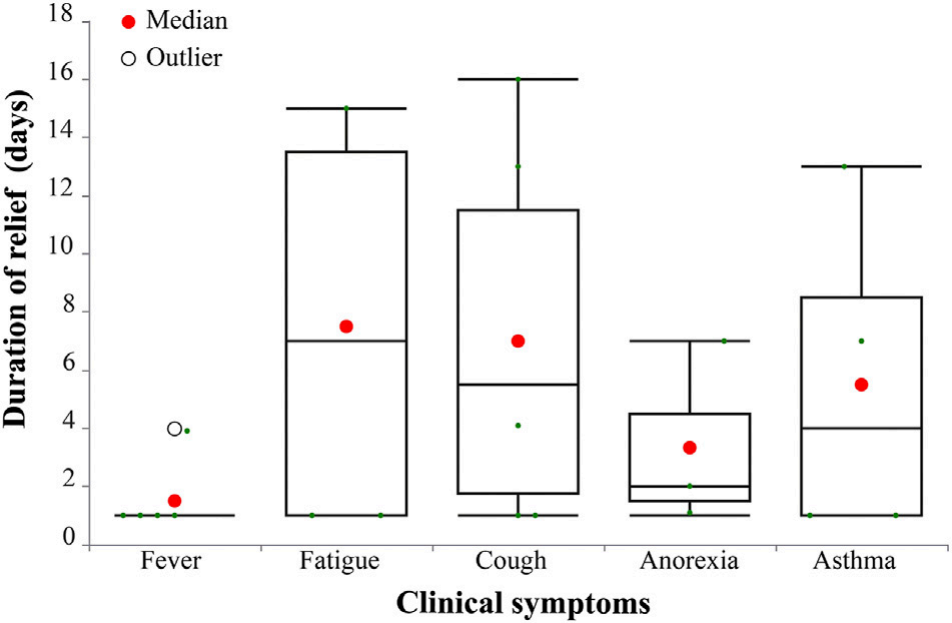
Duration of major clinical symptoms in eight COVID-19 cases (days).

**FIGURE 6:**
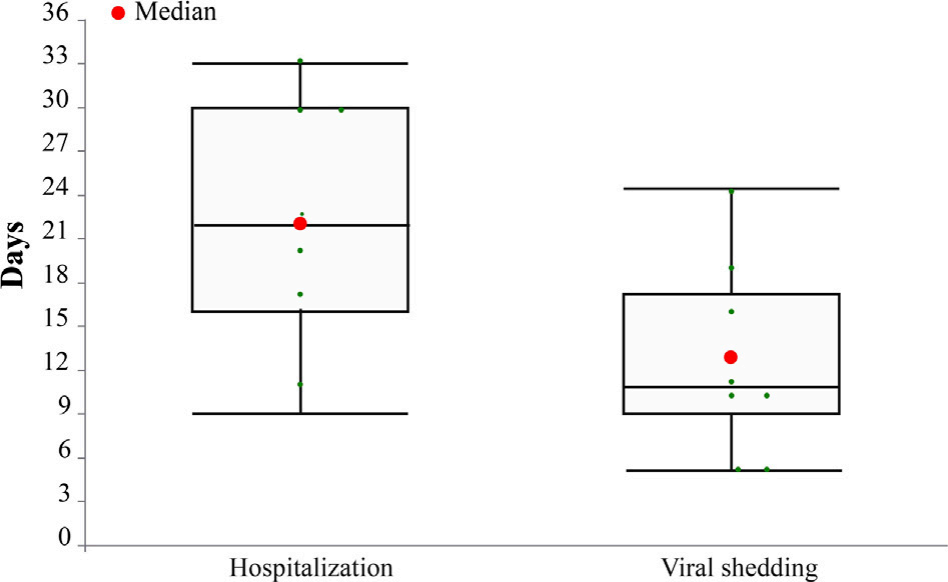
Duration of hospitalization and viral shedding in eight COVID-19 cases (days).

The median duration of fever recovery was 1 day, fatigue recovery was 7 (1.0, 13.5) days, cough recovery was 5 (3.0, 10.0) days, anorexia recovery was 2 (1.5, 4.5) days, and asthma recovery was 4 (1.0, 8.5) days (Figure 5). None of the eight patients experienced serious adverse reactions during hospitalization. The median durations of viral shedding and hospitalization were 10.5 and 21.5 days, respectively (Figure 6).

### Laboratory Examination and Radiologic Findings

Table 4 shows the main laboratory and radiological results for these patients. After two courses of QFPDD, abnormal laboratory indexes including neutrophil count, lymphocyte count, white blood cell count, lactate dehydrogenase, and C-reactive protein recovered, suggesting that the recovery of abnormal indicators was associated with the use of QFPDD.

**TABLE 4:** Laboratory and CT findings of eight COVID-19 cases before and after QFPDD treatment.

Variables	Case 1		Case 2		Case 3		Case 4		Case 5		Case 6		Case 7		Case 8	
	Day 0	Day 6	Day 0	Day 6	Day 0	Day 6	Day 0	Day 6	Day 0	Day 6	Day 0	Day 6	Day 0	Day 6	Day 0	Day 6
Laboratory findings																
NEU, ×10^9^/L (2.5–7.5)	0.57 (↓)	3.93	5	4	6.96	3.6	18.01 (↑)	9.62 (↑)	2.31 (↓)	5.3	2.38 (↓)	4.65	6.28	2.21 (↓)	5.99	4.72
LY, ×10^9^/L (0.8–4.0)	1.51	1.18	1.6	2.2	1.42	0.63 (↓)	0.66 (↓)	1.01	0.73	0.95	1.17	2.23	0.50 (↓)	1.16	1.2	1.27
WBC, ×10^9^/L (4.0–10.0)	6.67	6.03	7.1	6.8	9.25	5.67	19.07 (↑)	11.23 (↑)	3.3 (↓)	7	3.90 (↓)	7.64	7.6	4.37	7.8	6.73
PLT, ×10^9^/L (100–350)	155	NR	279	221	NR	170	143	NR	124	278	202	304	199	449 (↑)	276	251
AST, U/L (15–40)	18	17	16	15.3	19.8	16.4	30.5	23.4	39.2	36.2	39	21	11 (↓)	18	18	19
ALT, U/L (0–40)	14	15	15	15	27.7	20.3	19	23.9	7.2	11.2	29	31	21	22	16	20
ALP, U/L (female: 50–135; male: 45–125)	80.1	87.4	47.0 (↓)	54.9	78.3	77.1	52.4	47.7	38.9 (↓)	42.8 (↓)	43 (↓)	54	NR	NR	56	70
CK, U/L (18.0–198.0)	78	137	NR	77.8	NR	NR	120.5	NR	225	133	97	38	NR	NR	25	26
CK-MB, U/L (0–18)	32 (↑)	10	NR	NR	NR	NR	14	NR	0.9	1.2	10	8	NR	NR	8	10
LDH, U/L (135.0–215.0)	166	NR	143	153.4	NR	NR	254 (↑)	NR	781 (↑)	681 (↑)	376 (↑)	205	326 (↑)	NR	161	168
UREA, mmol/L (2.9–7.5)	7.76 (↑)	8.96 (↑)	4.6	5.22	NR	NR	4.36	NR	4.2	3.6	6.09	4.55	8.30 (↑)	5.8	5.16	5.06
CREA, mmol/L (female: 44–97; male: 54–106)	72.9	83.2	57	59.8	NR	NR	72.9	NR	77	78	72.4	57.4	75	77	61	61
PCT, mg/L (<0.5 mg/L)	0.1	0.23	0.064	NR	NR	NR	0.04	NR	0.05	<0.05	0.22	NR	0.06	0.05	NR	NR
ESR, mm/60 min (female: 0–20; male: 0–15)	12	12	44 (↑)	38 (↑)	NR	NR	NR	NR	NR	NR	42 (↑)	NR	40 (↑)	NR	NR	NR
CRP, mg/L (0.068–8.2)	<5	NR	18.12 (↑)	7.44	NR	2.9	31.92 (↑)	NR	0.85	1.25	40.3 (↑)	NR	10 (↑)	5	10.94 (↑)	4.9
CT funding																
No abnormal lesions	Yes	Yes	No	No	No	No	No	No	No	No	No	No	No	No	No	No
Unilateral lung injury	No	No	No	No	No	No	No	No	No	No	No	No	No	No	No	No
Bilateral lung injury	No	No	Yes	Yes	Yes	Yes	Yes	Yes	Yes	Yes	Yes	Yes	Yes	Yes	Yes	Yes

↑: higher than normal, ↓: lower than normal.

NR indicates not record data.

Neutrophil count (NEU), lymphocyte count (LY), white blood cell count (WBC), platelet count (PLT), aspartate aminotransferase (AST), alanine aminotransferase (ALT), creatine kinase (CK), creatine kinase isoenzyme (CK-MB), alkaline phosphatase (ALP), lactate dehydrogenase (LDH), urea nitrogen (UREA), serum creatinine (CREA), procalcitonin (PCT), erythrocyte sedimentation rate (ESR), and C-reactive protein (CRP) levels.

Of the eight patients, seven (87.5%) presented with bilateral macular or ground-glass lung lesions on admission, and one (12.5%) showed no abnormal lung lesions. After they received more than two courses of QFPDD, all seven patients showed different degrees of absorption (Table 4).

### Adverse Events

No adverse events were observed during the treatment. As required by *the Guidelines*, COVID-19 patients should revisit hospitals for a follow-up at 2 and 4 weeks after discharge from the hospital. During the follow-up period, no adverse events such as liver and kidney damage were observed in all eight cases.

## Discussion

In this pre-post study, eight patients were additionally offered QFPDD after group consultation by Western medicine and TCM doctors. QFPDD may facilitate the improvement of clinical symptoms such as fever, anorexia, cough, asthma, etc., and the absorption of pulmonary lesions improves. During hospitalization, none of the patients’ health worsened or died, and all were cured and discharged. No serious adverse events related to QFPDD were observed. The results indicated that QFPDD had favorable potential effectiveness and safety in treating COVID-19 with other respiratory diseases.

According to the theory of TCM, COVID-19, particularly in the cold season of late 2020 and early 2021 in mainland China, is mainly induced by cold, dry, and damp factors, which were seen as external pathogenic factors by TCM doctors. These external pathogenic factors originating outside the body can easily invade the lungs, causing respiratory diseases. QFPDD can relieve the interior and exterior of the body, unblock and regulate *sanjiao* (three burners in TCM, with the upper burner relating to organs in the thorax and the breathing function, the middle burner relating to the organs on top of the stomach and the digesting function, and the lower burner relating to the organs below the abdomen and the urogenital functions) (Fan et al., 2020), which means it could boost patients' immunity and promote the recovery of body functions. The herbs in QFPDD also promotes lung Qi, dispel pathological factors and relieve toxins, eliminate moisture and dampness, and purge heat by removing water (Liu et al., 2021). In addition, owing to the combination of other respiratory diseases, the clinical features of the patients are slightly different from those of ordinary patients with COVID-19. Studies have found that major clinical symptoms such as fever, cough, asthma, anorexia, and fatigue were improved after treatment with QFPDD (Meng et al., 2020; Sun et al., 2020). The curative effect of this prescription might be related to its composition.

The theory of QFPDD can be traced back to one of the classical TCM works, *Treatise on Cold Pathogenic and Miscellaneous Diseases*. QFPDD comprises 21 herbs, which can be divided into four formulas: 1) Ma Xing Gan Shi decoction, which can clear lung Qi and relieve symptoms of asthma, and has a positive effect on the symptoms of fever and cough; 2) Shegan Mahuang decoction, which can warm the lungs, resolve phlegm, and relieve phlegm and asthma symptoms; 3) Minor decoction of Bupleurum, which can harmonize and relieve Shaoyang, and improve the metabolic capacity of the human body; and 4) Wu Ling decoction, which can warm yang and damp drains. It has a diuretic effect. The combined effect of these herbs could thus relieve patients’ cough, fever, nasal obstruction, sore throat, and other symptoms significantly compared with before the use of QFPDD. The combination with QFPDD could significantly shorten the duration of symptom improvement according to existing research (Wang et al., 2020).

Studies have shown that the components of QFPDD have a certain theoretical basis for improving the symptoms of other respiratory diseases in TCM. According to experienced TCM doctors, the basis of COPD and bronchiectasis is phlegm, heat, static blood, and insufficiency. In the acute attack period of the disease, Ma Xing Gan Shi decoction can be used to clear lung heat, which can be regarded as a representative prescription to relieve cough and asthma to some extent. In addition, the use of QFPDD in the treatment of bacterial pneumonia has been reported (Zheng and Li, 2010). Therefore, the pathogenesis of TCM and the pathological basis of COVID-19 are similar to those of the aforementioned diseases, and overlaps exist in the application of prescriptions. Thus, QFPDD may be effective in alleviating COPD, bronchiectasis, bacterial pneumonia, and other respiratory diseases. However, further research is required to support our findings.

Related studies have shown that patients with COVID-19 with other respiratory diseases, particularly COPD, are more likely to have severe outcomes than common patients with COVID-19 (Sanchez-Ramirez and Mackey, 2020), with a longer course of the disease and a higher mortality rate (Lohia et al., 2021), which may be due to damage to the immune system caused by other diseases of the respiratory system (Chakraborty et al., 2020). However, in this observational study, patients had good curative outcomes and prognosis, which may be related to the addition of QFPDD.

Clinical studies have confirmed that the main clinical symptoms, such as fever, cough, asthma, and fatigue, were significantly improved after treatment with QFPDD, and the symptoms of fever, cough, fatigue, and headache improved faster than before (Zhu et al., 2020; Wang et al., 2020). Studies have identified the potential mechanism of QFPDD in the treatment of COVID-19, mainly by acting on key molecules such as interleukin, mitogen-activated protein kinase, and TNF (tumor necrosis factor), affecting the toll-like receptor pathway, TNF signaling pathway, and interleukin signaling pathway, exerting immunomodulatory, anti-infection, and anti-inflammatory storm effects (Niu et al., 2020; Niu et al., 2021).

In terms of improving clinical symptoms, we found that the clinical features of these patients were slightly different from those of common patients with COVID-19. The main clinical symptoms of COVID-19 are fever, cough, and fatigue (The NHC of the People’s Republic of China, 2020). This may be due to a combination of other diseases of the respiratory system.

Moreover, we found that QFPDD effectively relieved the clinical symptoms of patients with other respiratory diseases, such as fever and cough. The lung lesions in these patients showed varying degrees of absorption. During the two courses of the treatment, the number of patients with major clinical symptoms showed a general decreasing trend. Most of the hospitalized symptoms completely disappeared after two courses of decoction.

Related studies have shown that morbidity and mortality rates of COVID-19 are higher in older people than in younger people, with a mortality rate of 80% in people aged more than 60 years, particularly in those with underlying medical conditions (Li et al., 2020; Zhavoronkov, 2020). Notably, in this study, all patients who were elderly and aged 60 years and more were cured and discharged, and no deaths occurred. Wang Q. et al. (2021)reported that QFPDD combined with Western treatment demonstrated favorable outcomes including improving clinical symptoms and promoting the absorption of lung lesions in elderly COVID-19 patients. We also observed improvements in white blood cell count, lymphocyte count, C-reactive protein, lactate dehydrogenase, and creatine kinase after QFPDD administration, which is consistent with previous studies (Xin et al., 2020).

In this study, we concluded that QFPDD might help improve the clinical symptoms of COVID-19 patients with other respiratory diseases; no serious adverse events related to QFPDD were observed, which was in line with previous studies (Wang J. et al, 2021; Wang Q. et al, 2021; Chen et al., 2021; Zhang et al., 2021; Zhang et al., 2022). The results of this study may provide a reference for clinicians to use QFPDD treatment. In addition, research on QFPDD is increasing and can prove its potential effectiveness. Early treatment with QFPDD is closely related to improving the clinical recovery rate and shortening the acid negative status, hospital stay, and course of disease (Shi et al., 2020). Multiple retrospective studies have shown that this decoction can halve the risk of death in patients hospitalized with COVID-19 (Zhang et al., 2021) and enhance the immune response against COVID-19 by detecting blood samples after TCM treatment (Luo et al., 2020). It can also exhibit the effects of immune regulation, anti-infection, anti-inflammation, and multi-organ protection (Zhao et al., 2021). These studies may explain the favorable prognosis of the patients in this study.

However, this study has two main limitations. First, this was a retrospective study with small sample size, and the data were collected at the time of emergency treatment, leading to the conclusion that some data were not collected. Second, no parallel control group was used in this study. In the future, large-sample, multi-center, randomized controlled studies will be conducted to further clarify the clinical efficacy of QFPDD in the treatment of COVID-19 combined with other diseases of the respiratory system.

## Data Availability

The original contributions presented in the study are included in the article/Supplementary Material; further inquiries can be directed to the corresponding author.
